# Irregular and bursty neuronal firing extend spike-timing dependent plasticity window to behavioral timescales

**DOI:** 10.1016/j.isci.2026.117052

**Published:** 2026-08-04

**Authors:** Yulia Dembitskaya, Silvana Valtcheva, Yihui Cui, Zhiwei Zheng, Srdjan Ostojic, Michael Graupner, Laurent Venance

**Affiliations:** 1 Center for Interdisciplinary Research in Biology (CIRB), College de France, CNRS, INSERM, Université PSL, Paris, France; 2 Department of Neurobiology and Department of Neurology of Sir Run Run Shaw Hospital, Zhejiang University School of Medicine, Hangzhou 310058, China; 3 Laboratoire de Neurosciences Cognitives et Computationnelles, INSERM U960, Ecole Normale Superieure – PSL Research University, Paris, France; 4 Université Paris Cité, CNRS, SPPIN – Saints-Pères Paris Institute for the Neurosciences, Paris, France

**Keywords:** synaptic plasticity, spike-timing-dependent plasticity, naturalistic patterns, Striatum, Cortex, patch-clamp, Mathematical modeling, irregular spiking, memory, behavioral timescale

## Abstract

Synaptic plasticity, which is thought to underlie learning and memory, is commonly induced in experimental settings with regular activity patterns. However, such regularity strongly differs from natural *in vivo* firing statistics. Therefore, it remains unclear how *in vivo*-like patterns, such as *irregular* and *bursty activity*, shape synaptic plasticity. We combined mathematical modeling and *ex vivo* patch-clamp experiments inducing naturalistic spike-timing-dependent plasticity (STDP) at cortico-striatal synapses. We found that irregular spike-pair stimulation diminished LTD occurrence and widened the LTP temporal window compared to regular patterns at low firing rate. Furthermore, increasing the firing rate abolished LTD to the profit of LTP. Our modeling and experimental data indicate that bursts of action potentials are key contributors to this effect. These results highlight the importance of naturalistic firing statistics and show that irregular and bursty firings extend the typical compressed STDP expression temporal window toward timescales relevant for behavior.

## Highlights

• A model-driven experimental approach reveals STDP rules under naturalistic activity
• Irregular firing reduces LTD occurrence and broadens the temporal window for LTP
• Bursts account for much of the effect of irregular activity on STDP expression
• Irregular and bursty firing extend STDP timescales to behaviorally relevant regimes

## Graphical abstract

**Figure:**
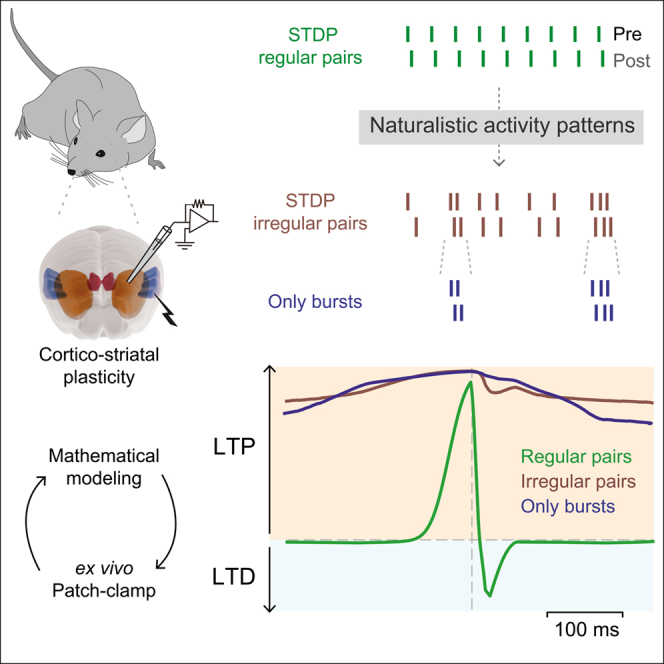

## Teaser

Neuroscience; Systems neuroscience; Cellular neuroscience

## Introduction

Understanding the mechanisms of synaptic plasticity is crucial for elucidating the principles of learning and memory in the brain.^1^ To emulate processes occurring *in vivo*, various *in vitro* and *ex vivo* cell-conditioning protocols have been devised and spike-timing-dependent plasticity (STDP), a synaptic Hebbian learning rule,^2^ emerged as a candidate synaptic mechanism to account for experience-driven modifications in neuronal circuits.^3^^,^^4^^,^^5^^,^^6^^,^^7^ STDP studies have primarily relied on regular induced activity patterns, which do not fully replicate the complexity of *in vivo* neuronal firing. Experimental studies *in vitro* and *ex vivo* preparations have shown that the induction of synaptic long-term potentiation (LTP) and depression (LTD) depends on (*i*) the firing rates of pre- and postsynaptic neurons^8^^,^^9^ and on (*ii*) the precise timing of pre- and post-synaptic action potentials.^3^^,^^4^^,^^6^^,^^10^^,^^11^^,^^12^^,^^13^^,^^14^^,^^15^ However, it remains unclear how firing rate and precise spike timing interact under more naturalistic activity patterns, and whether one mechanism predominates. Important difference lies in the firing statistics: in the majority of experimental protocols *ex vivo*, the imposed spike trains are regular and the relative timing between every pre- and post-synaptic spike (Δt) is constant. By contrast, natural firing patterns recorded *in vivo* are generally quite irregular as observed in cortical pyramidal cells firing^16^^,^^17^ and the timing between pre- and post-synaptic spikes varies.^18^^,^^19^^,^^20^ In addition to this irregularity, natural activity frequently contains bursts of action potentials, which strongly impact calcium dynamics and plasticity induction.^21^^,^^22^

Several experimental studies have investigated cortical plasticity in response to irregular firing patterns.^15^^,^^23^ In a spike-pair protocol where pre- and postsynaptic spikes were jittered, low presentation rates induced LTD, while high rates induced LTP in synapses between layer V neurons in the rat visual cortex.^9^ In the same line and still in the rat visual cortex, *in vivo* jittered presynaptic inputs account for the specific Hebbian STDP properties observed *in vivo* when compared to the corresponding *ex vivo* STDP.^20^ Repeated presentations of 1-s-long spike trains, recorded *in vivo* from L2/3 neurons in the rat visual cortex with partially overlapping receptive fields, triggered plasticity predictable by the spike-timing relationships between pre- and postsynaptic spikes.^24^ Additionally, replaying action potential time series recorded simultaneously from hippocampal place cells in freely moving rats at CA1 synapses induced LTP.^25^ In addition, using a lever press task in mice, STDP-like mechanisms spontaneously occurred *in vivo* in pyramidal cells of the motor cortex.^26^ These experiments indicate that STDP might occur under natural conditions.^6^^,^^15^^,^^23^^,^^27^^,^^28^ However, the exact role of spike timing versus firing rate in driving synaptic plasticity is still debated.^29^^,^^30^

Previous investigations using various synaptic plasticity computational models and naturalistic irregular firing patterns have demonstrated that synaptic changes induced by precise action potential timing are significantly weaker than those triggered by regular stimulation.^31^ Consequently, the temporal window of STDP expression and the conditions for its emergence under irregular activity remains to be better determined. Defining this window is particularly important for linking synaptic plasticity induction mechanism (sub-second) to behavioral (several seconds) timescales,^32^^,^^33^^,^^34^^,^^35^^,^^36^^,^^37^^,^^38^^,^^39^ which remains a neurophysiological conundrum.^23^^,^^40^^,^^41^

Here we use model-driven experiment strategy to delineate the expression domain of STDP under naturalistic conditions. We fit a phenomenological calcium-based model^31^^,^^42^ to plasticity recordings induced by regular paired activity on either side of the cortico-striatal synapse. We then predict how irregular activity shapes the spike-timing dependence of synaptic changes as a function of the firing rate and experimentally test these predictions using *ex vivo* patch-clamp recordings. In particular, we examine how firing rate, spike timing and burst firing interact to shape the expression of STDP to explore how naturalistic activity patterns can extend plasticity mechanisms toward behavioral timescales. We showed that bidirectional STDP is restricted to sparse or low activity regimes when using irregular paired stimulation. Increases in firing rate or bursts diminish progressively spike-timing influence and lead to plasticity regimes dominated by unidirectional plasticity, i.e., LTP. By dissecting the effects of firing rate and spike-timing, our approach identifies activity patterns that are effective in inducing plasticity during learning *in vivo*.

## Results

### LTP dominance upon increasing frequency of regular spike-pair stimulation in the dorsolateral striatum

**Figure 1:**
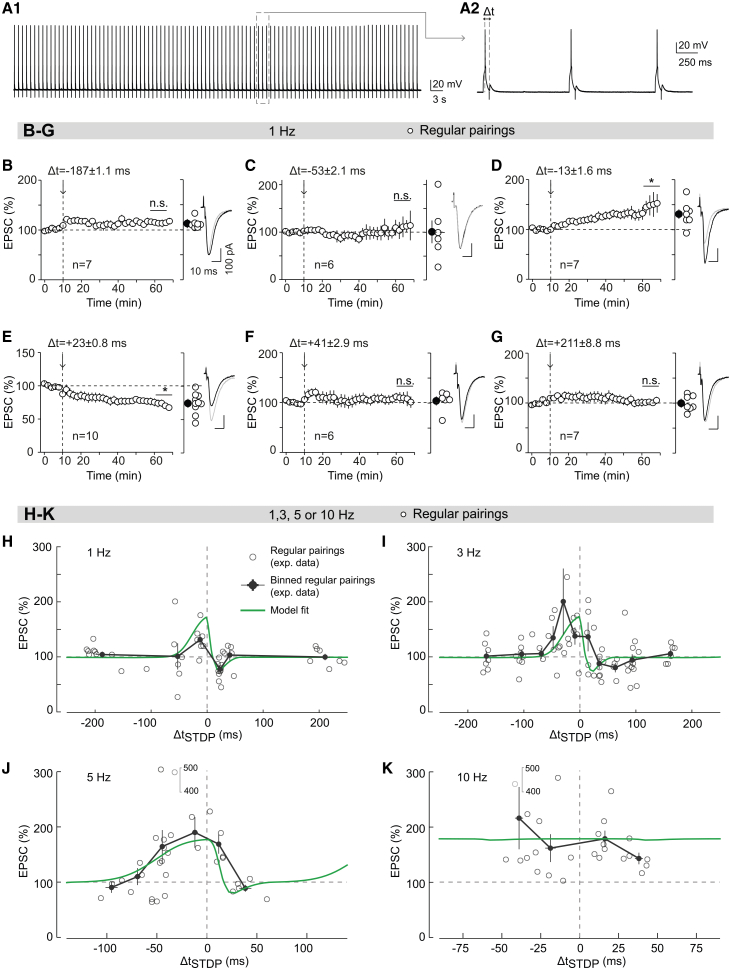
LTP dominance upon increasing frequency (1–10 Hz) for regular spike-pair stimulation in the dorsolateral striatum (A) Postsynaptic membrane potential recording during STDP induction with regular pairings (Δt and *f* being kept fixed) including an evoked single postsynaptic spike paired with a single cortical stimulation (the zoom view shows the stimulation artifact on three pairings); this pairing is repeated 100 times at *f* = 1 Hz. (B and C) Averaged time-courses showing the absence of long-term plasticity for large negative values of Δt, such as (B) Δt = −187 ± 1.1 ms (112.3 ± 3.7%, *n* = 7, *p* = 0.1479) and Δt = −53 ± 2.1 ms (100.1 ± 2.4%, *n* = 6, *p* = 0.9707). (D and E) Expression of STDP for restricted values of Δt: (D) LTP induced by post-pre pairings (for Δt = −13 ± 1.6 ms, 131.3 ± 9.6%, *n* = 7, *p* = 0.0171) and (E) LTD induced by pre-post pairings (for Δt = +23 ± 0.8 ms, 74.4 ± 4.9%, *n* = 10, *p* = 0.0005). (F and G) Absence of long-term plasticity for large positive values of Δt, such as (F) Δt = +41 ± 2.9 ms (104.3 ± 8.2%, *n* = 6, *p* = 0.6228) and (G) Δt = +211 ± 8.8 ms (99.7 ± 5.9%, *n* = 7, *p* = 0.9592). In the summary plots to the right of each time course, full black dots represent the average of all STDP experiments and each point (empty black circles) represents the percentage of change in excitatory postsynaptic current (EPSC) amplitude at 50–60 min after STDP pairings in a single STDP experiment. Transients display the average EPSC amplitude at baseline (gray) and at 50–60 min after STDP pairings (black). Error bars represent the SEM. ns, not significant; ∗, *p* < 0.05 by one sample *t* test. (H–K) Summary graphs of STDP observed at 1 (H), 3 (I), 5 (J), and 10 (K) Hz. Each data point (gray open circle) corresponds to the average synaptic change of one regular spike-pair experiment with a fixed time lag, Δt, and corresponding stimulation frequency (*Methods*). Full black circles denote mean and SEM of the binned experimental data. Green line, synaptic change from the calcium-based plasticity model fitted to binned data points. The frequency of the regular spike-pair stimulation is given on top of each figure. All plasticity results are shown for 100 pairings.

We investigated the effect of different stimulation patterns on STDP in striatal projecting neurons (SPNs) of the dorsolateral striatum using horizontal brain slices from rats. We first explored STDP with regular paired stimulation at *f* = 1 Hz (Figure 1A).^43^^,^^44^^,^^45^ Following classical STDP protocols,^6^ we paired the pre- and postsynaptic stimulations with timing between pre- and post-synaptic activation (Δt) and a constant number of pairs (*f*). Δt <0 indicates that the post-synaptic stimulation occurred before the paired pre-synaptic one (post-pre pairings) and Δt >0 indicates that the pre-synaptic stimulation occurred before the post-synaptic one (pre-post pairings). In these conditions, we observed a bidirectional and asymmetric anti-Hebbian STDP confined within short Δt (∼60 ms) (Figures 1B–1G). Indeed, we observed LTP for Δt ∼ –20 ms (Δt = −13 ± 1.6 ms, *p* = 0.0171, *n* = 7; see representative STDP in Figure S1A1 and the overall plasticity in Figures 1D and 1H), and LTD for Δt∼+20 ms (Δt = +23 ± 0.8 ms, *p* = 0.0005, *n* = 10; see representative STDP in Figure S1B and the overall plasticity in Figures 1E and 1H). For larger Δt, i.e., −50 ms and +40 ms (Figures 1C and 1F) and ±200 ms (Figures 1B and 1G), no plasticity could be induced (Δt = −53 ± 2.1 ms or +41 ± 2.9 ms: *p* = 0.9707, *n* = 6, and *p* = 0.6228, *n* = 6, Figures 1C and 1F, respectively; Δt = −187 ± 1.1 ms or +211 ± 8.8 ms: *p* = 0.1479, *n* = 7, and *p* = 0.9592, *n* = 7; Figures 1B and 1G, respectively; see representative STDPs in Figures S1C and S1D). This is in line with the anti-Hebbian cortico-striatal STDP previously reported in native conditions, i.e., without blocking GABAergic transmission.^43^^,^^45^^,^^46^ Note that under inhibition of ionotropic GABAergic transmission, cortico-striatal Hebbian STDP can be observed^47^^,^^48^ due to the role of tonic GABA which acts as a developmental Hebbian/anti-Hebbian switch^46^^,^^49^ in both rats and mice. Here, we thus performed cortico-striatal STDP recordings without GABA_A_R antagonist to preserve the striatal microcircuits and the anti-Hebbian polarity as observed *in vivo* in adult rats.^50^^,^^51^ Anti-Hebbian, although less widespread than Hebbian STDP, has been identified in various structures (hippocampus, striatum, cerebellum and brainstem) and neuronal subpopulations (see the pioneering STDP work of Bell et al.^10^ in the electrosensory system of the mormyrid fish),^6^ and theoretical studies proposed that anti-Hebbian STDP might allow for the learning of sequences of spikes at cortico-striatal synapses^52^ or stabilizing replay dynamics in hippocampus.^53^

The frequency at which pairings are presented (*f*) has a strong impact on STDP expression.^4^^,^^6^^,^^9^^,^^11^^,^^16^^,^^32^^,^^45^^,^^54^^,^^55^ We thus investigated the expression of cortico-striatal STDP as a function of firing rate (Figures 1H–1K). We explored a frequency range compatible with *in vivo* striatal recordings in awake adult rats, i.e., 1–6 Hz (see e.g.,^56^^,^^57^^,^^58^^,^^59^). When increasing the pair presentation frequency *f* from 1 to 10 Hz, we observed that the STDP temporal window was enlarged and pre-post LTD progressively disappeared giving way to LTP, leading to a symmetric LTP at 10 Hz (STDP with *f* = 1 Hz, *n* = 43; *f* = 3 Hz, *n* = 32; *f* = 5 Hz, *n* = 30; and *f* = 10 Hz, *n* = 25 SPNs; Figures 1H–1K). In particular, asymmetrical STDP around Δt = 0 ms was observed up to 3 Hz (Figures 1H and 1I), and then LTP overtook the pre-post pairing domain (at 5 Hz; Figure 1J) to become the only form of plasticity regardless the Δt values (at 10 Hz; Figure 1K).

In conclusion, STDP with regular spike-pair simulation at low rate (1–3 Hz) in the dorsolateral striatum displayed anti-Hebbian asymmetric plasticity—LTP for post-pre pairings and LTD for pre-post pairings—within a narrow temporal window spanning around 60 ms (−30 < Δt < +30 ms) (Figure 1H). The shape of the STDP window tightly depends on the frequency of spike-pair presentations, giving rise to a symmetric plasticity rule with LTP dominating at elevated frequencies (*f* ≥ 5 Hz) (Figures 1J and 1K).

### Calcium-based plasticity model reproduces spike-timing and -frequency dependence

We next examined whether a simple plasticity model can account for the main features of experimental cortico-striatal STDP data obtained with regular spike-pair stimulation at different stimulation frequencies. We used a phenomenological calcium-based model, which describes in a simplified manner the signaling cascades that underlie synaptic potentiation and depression.^31^^,^^42^ In that model, synaptic changes are driven by pre- and postsynaptic spikes inducing calcium transients of different amplitudes. The compound calcium trace in turn triggers depression and/or potentiation when the respective depression and/or potentiation thresholds are crossed making the model naturally sensitive to spike-timing and rate.^31^^,^^42^ The model relies on parameters describing the calcium dynamics and how depression/potentiation threshold crossing update that synaptic weight (in total 12 parameters) that can be directly fit to experimental data (nine parameters were optimized during the fitting routine; *Methods* and Table 1). Considering constant experimental conditions across the datasets obtained at different stimulation frequencies, we require that a single set of model parameters accounts for the data across all tested spike-pair frequencies.

**Table 1:** Parameters obtained from fitting the calcium-based plasticity model to experimental data from regular spike-pairings

Parameter	Value
τ_Ca_ (ms)	66.718
*C*_pre_	1.45248
*C*_post_	0.405039
θ_d_	1^a^
θ_p_	1.5^a^
γ_d_	2.0
γ_p_	15.973
σ	0.3
τ (s)	16.3457
*D* (ms)	−1.56591
β	0.5^a^
*b*	8.28729

a These parameters were fixed and were not optimized during the fitting procedure (see Results).

Fitting the model, we find that all the features of the cortico-striatal plasticity experiments with regular stimulation at 1, 3, 5, and 10 Hz (Figures 1H–1K) were well reproduced. Specifically, the model correctly produces the occurrence of potentiation at negative Δt and depression at positive Δt at frequencies of 1 and 3 Hz. A previous study examined how combinations of parameters determine the shape of the resulting STDP curve according to depression and/or potentiation occurring along the Δt axis.^42^ In agreement with this study, the resulting parameter set is within the potentiation-depression region of possible STDP curves—first potentiation and then depression when going along the Δt axis. Potentiation-depression curves occur when the presynaptically induced calcium amplitude is larger than the potentiation threshold and the postsynaptically induced calcium amplitude (i.e., $C_{post}<\theta_{p}<C_{pre}$). Furthermore, the model captures the disappearance of depression at 5 Hz stimulation (Figure 1J) and constant potentiation—no matter the time lag, Δt, for 10 Hz stimulation. The frequency-dependence emerges naturally in the model when calcium transients of consecutive spike-pairs start to interact with each other and the time spent above depression/potentiation thresholds is changed in consequence. This interaction becomes significant at stimulation frequencies comparable to the calcium decay time constant ($\tau_{Ca}$). $\tau_{Ca}$ is selected during the fitting process to capture the effect of stimulation frequency on the STDP curve. In other words, the decay time constant is tuned during model fitting to account for the frequency-dependent changes observed in the STDP curve (Figures 1H–1K, with green lines for the model fit).

In summary, the calcium-based plasticity model reproduced qualitatively and quantitatively the experimental cortico-striatal plasticity data. The disappearance of LTD at intermediate frequencies and the dominance of LTP at high frequencies emerge directly from the model’s intrinsic frequency dependence.

### Irregular spiking promotes LTP expression in an extended Δt range

With parameters constrained by a set of experimental data on regular Δt spike-pair stimulation (Figures 1H–1K), the plasticity model was next used to predict outcomes for irregular protocols under the same conditions. We explored plasticity induced by spike-pairs with a fixed time lag Δt between pre- and post-synaptic spikes, but with an irregular temporal occurrence of these spike-pairs. Specifically, spike pairs were generated according to a Poisson process, such that the intervals between successive pairings varied stochastically. As a result, each repetition of the protocol in the simulations and the experiments consisted of a different realization of the same stochastic process, with identical statistical properties (mean rate and fixed Δt) but a distinct temporal sequence of spike-pairs. Irregular Δt spike-pair stimulation has been shown theoretically to strongly affect the shape of the STDP curve by reducing LTD and favoring LTP when compared to regular spike-pair stimulation at the same frequency.^31^

**Figure 2:**
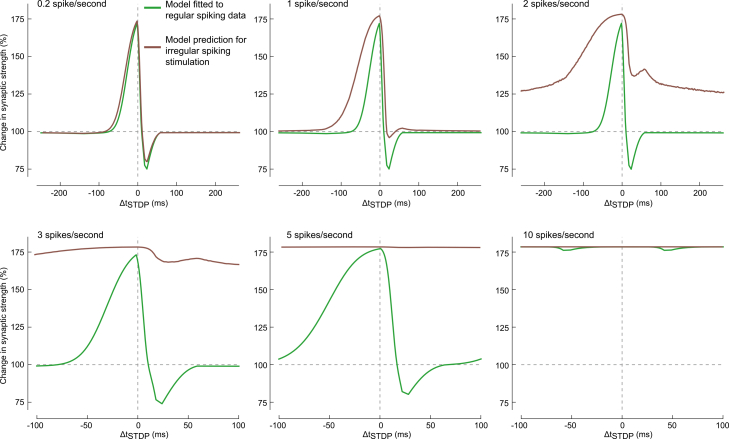
Model fit of STDP for regular spike pairs and prediction for irregular spike pairs at various activity rates The change in synaptic strength as a function of the time-lag between pre- and postsynaptic spikes, Δt, is shown for six different stimulation rates: 0.2, 1, 2, 3, 5, and 10 spikes/second (indicated at the top of each figure). Green line, fit of the calcium-based plasticity model to the experimental data obtained with regular spike-pair stimulation (from Figures 1H–1K). Brown line, prediction of the model for irregular spike-pair stimulation. In the irregular condition, spike-pairs were generated according to a Poisson process, resulting in stochastic intervals between successive pairings; each simulation corresponds to a different realization of this process with identical mean rate and fixed Δt. All results are shown for 100 spike pairs presented regularly (green) or irregularly (brown).

We compared synaptic changes induced by irregular spike-pairs to those induced by regular spike-pairs (at 0.2, 1, 2, 3, 5, and 10 spikes/second) using the cortico-striatal parameter set in the model (Figure 2). The model predicts two important distinctions: (1) at low stimulation rates (one spike/second), the range of time lags Δt leading to LTP is extended for negative Δt values, while LTD range is reduced and nearly vanishes at positive Δt for irregular stimulation (Figure 2); and (2) at two and three spikes/second, irregular stimulation produces robust LTP across all Δt values, abolishing the timing dependence observed under regular stimulation (Figure 2). These two effects have been described as general properties, independent of the plasticity model.^31^ Interestingly, at low frequencies (0.2 spike/second), an irregular stimulation does not significantly affect plasticity expression compared to a regular stimulation, due to the short temporal integration window given by the calcium decay time constant (τ ≈ 66.7 ms). As the stimulation frequency increases, irregular stimulation progressively deviates from regular stimulation. This is due to the increasing probability of short inter-spike intervals, which allows calcium transients from different spike pairs to overlap and interact. This effect becomes noticeable already at one spike/second and strengthens with higher frequencies (2–10 spikes/sec), leading to the gradual divergence between regular and irregular stimulation (Figure 2). In summary, when shifting from regular to irregular spike-pair stimulation—and keeping all other aspects in the paired activity fixed—calcium-based plasticity models predict that irregular patterns trigger strong changes of the STDP expression curve resulting in an LTP dominance and a diminished dependence on spike-timing, in term of Δt values, as stimulation frequency increases (Figure 2).

**Figure 3:**
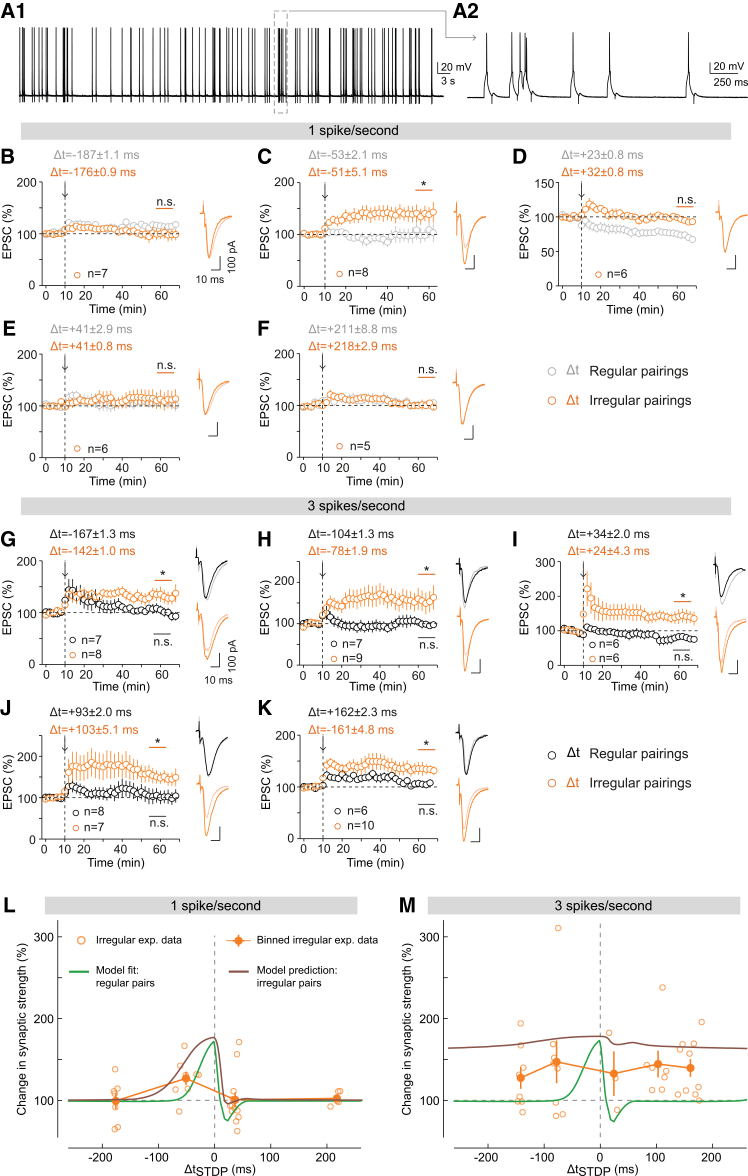
Cortico-striatal plasticity for irregular spike-pair stimulation: experimental data and model predictions (A) Postsynaptic membrane potential recording during an STDP protocol with irregular pairings, i.e., fixed Δt and irregular Poisson-distributed inter-pairing intervals, with an evoked-postsynaptic single spike paired with a single cortical stimulation (see zoom-in on 2.5 s of the recording, A2). Irregular spike pairs where repeated at 1 (B–F) or 3 (G–K) spikes/second, with the number of pairings fixed at 100. (B–F) Averaged time-courses of synaptic weights after one spike/second post-pre or pre-post regular (gray circles; data for regular pairing STDP at 1 spike/second are taken from Figures 1B–1G) or irregular (orange circles) pairings for various Δt (B, Δt = −176 ± 0.9 ms: 99.4 ± 10.0%, *p* = 0.9541, *n* = 7; C, Δt = −51 ± 5.1 ms: 126.9 ± 8.3%, *p* = 0.0143, *n* = 8; D, Δt = +32 ± 0.8 ms: 96.2 ± 3.6%, *p* = 0.4486, *n* = 6; E, Δt = +41 ± 0.8 ms: 105.3 ± 18.9%, *p* = 0.7917, *n* = 6; F, Δt = +218 ± 2.9 ms: 102.41 ± 3.7%, *p* = 0.9712, *n* = 5). Transients to the right display the average EPSC amplitude at baseline (pale color) and at 50–60 min after STDP pairings (full color). (G–K) Averaged time-courses of plasticity after 3 spikes/second stimulation with regular- (black circles) or irregular pairs (orange circles). For regular pairings, LTP was not observed at all tested Δt (G, Δt = −167 ± 1.3 ms: 101.3 ± 9.8%, *p* = 0.8972, *n* = 7; H, Δt = −104 ± 1.3 ms: 101.4 ± 10.0%, *p* = 0.8918, *n* = 7; I, Δt = +34 ± 2.0 ms: 80.0 ± 9.3%, *p* = 0.0877, *n* = 6; J, Δt = +93 ± 2.0 ms: 100.4 ± 15.8%, *p* = 0.9826, *n* = 8; K, Δt = +162 ± 2.3 ms: 105.8 ± 6.8%, *p* = 0.4284, *n* = 6). With irregular pairings with 3 spikes/second, LTP was observed at all tested Δt (G, Δt = −142 ± 1.0 ms: 130.5 ± 12.5%, *p* = 0.0452, *n* = 8; H, Δt = −78 ± 1.9 ms: 132.4 ± 13.1%, *p* = 0.0423, *n* = 9; I, Δt = +24 ± 4.3 ms: 145.1 ± 15.5%, *p* = 0.0332, *n* = 6; J, Δt = +103 ± 5.1 ms: 144.4 ± 16.3%, *p* = 0.0342, *n* = 7; K, Δt = +161 ± 4.8 ms: 139.6 ± 10.9%, *p* = 0.0055, *n* = 10). (L) Summary graphs comparing model predictions of synaptic changes with experimental data for irregular spike-pairs as a function of the time lag, Δt, at 1 spike/second stimulation. Individual experimental data points (orange empty circles) and the binned data (orange full circles and line) summarize the experiments in (B–F). Model fit for regular pairs (green line) and prediction for irregular pairs (brown line). (M) Same depiction as in L but for 3 spikes/second irregular spike-pair stimulation. Each circle represents the percentage of change in EPSC amplitude at 50–60 min after STDP pairings in a single STDP experiment. Error bars represent the SEM. ns, not significant; ∗, *p* < 0.05 by one sample *t* test.

We then tested experimentally the model predictions by constructing stimulation protocols from Poisson-distributed spike-pairs with fixed Δt (from −200 to +200 ms; see *Methods* for details). Each experimental stimulation protocol corresponded to a different realization of this stochastic process, with identical statistical properties but distinct temporal sequences of spike-pairs. We imposed this irregular activity pattern on pre- and postsynaptic neurons at two firing rates, 1 and 3 spikes/second (Figure 3A). A 1 spike/second—irregular—activity, in full agreement with model predictions, produced two main effects compared with regular stimulation: (i) an extension of the LTP domain toward more negative Δt values, and (ii) a reduced occurrence of LTD at positive Δt (Figures 3B–3F and 3L). Indeed, LTP was observed for Δt ∼ –50 ms (Δt = −51 ± 5.1 ms, *p* = 0.0143, *n* = (8) with irregular pairings, while no plasticity was detected after regular spiking (*p* = 0.9707, *n* = (6) (Figure 3C). For Δt∼+20–30 ms, irregular pairings failed overall to evoke any long-term changes (Δt = +32 ± 0.8 ms *p* = 0.3409, *n* = 6), while regular pairings induced LTD (*p* = 0.0005, *n* = 10) (Figure 3D). No significant plasticity was observed for larger negative or positive Δt (Δt = +41 ± 0.8 ms: *p* = 0.7917, *n* = 6; Δt = −176 ± 0.9 ms: *p* = 0.9541, *n* = 7; and Δt = +218 ± 2.9 ms: *p* = 0.5494, *n* = (5) (Figures 3B, 3E and 3F).

At 3 Hz (Figures 3G–3K and 3M), upon regular pairings LTP was not observed at any of the tested Δt values (Δt = +34 ± 2.0 ms: *p* = 0.0877, *n* = 6; Δt = −104 ± 1.3 ms: *p* = 0.8918, *n* = 7, Δt = −93 ± 2.0 ms: *p* = 0.9826, *n* = 7, Δt = −167 ± 1.3 ms: *p* = 0.8971, *n* = 7, and Δt = +162 ± 2.3 ms: *p* = 0.4284, *n* = (6) (Figures 3G–3K). Upon irregular pairings at 3 spikes/second, as predicted by the model, LTP was observed across all tested Δt without noticeable difference in plasticity amplitude in the range −150 < Δt< +180 ms (Δt = +24 ± 4.3 ms: *p* = 0.0332, *n* = 6; Δt = −78 ± 1.9 ms: *p* = 0.0423, *n* = 9; Δt = +103 ± 5.1 ms: *p* = 0.0342, *n* = 7; Δt = −142 ± 1.0 ms: *p* = 0.0452, *n* = 8, and Δt = +161 ± 4.8 ms: *p* = 0.0055, *n* = 10) (Figures 3G–3K and 3M).

**Figure 4:**
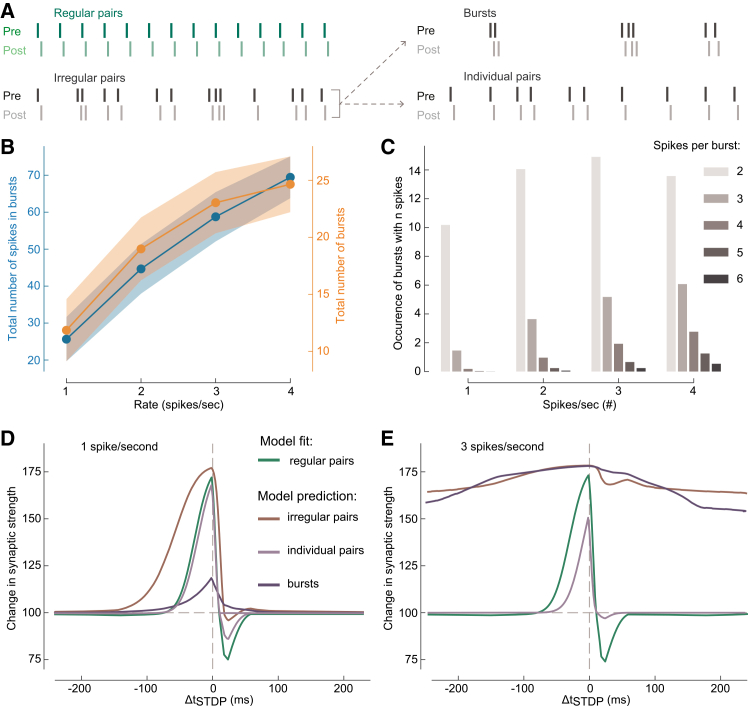
Model prediction for synaptic plasticity induced with bursts or individual pairs (A) Illustration of regular (dark and pale green lines) and irregular (black and gray lines) STDP stimulation protocols with the extraction of bursts and individual pairs. Short stimulation protocol periods are shown here, while 100 spike-pairs in total are used with regular- and irregular stimulation in this study. The irregular spike-pair stimulation is used to construct the burst stimulation by keeping only bursts of pre-post pairs; or the individual pre-post pair stimulation by eliminating all spikes occurring in bursts (marked in gray; see text for details). (B) Total number of spikes and number of bursts in the pre-post burst stimulation as function of stimulation rate (mean ± STD). (C) Number of spikes occurring in individual bursts for four different stimulation rates. Data shown in (B) and (C) are averages constructed from Poisson spike trains of the indicated rate and 100 spike-pairs (see *Methods* for details). (D and E) Change in synaptic strength prediction of the calcium-based plasticity model for pre-post burst and individual pre-post pair stimulation with a rate of 1 spike/second (D) and 3 spikes/second (E). Synaptic change prediction for burst stimulation is shown in dark blue, and for individual pairs in light purple. The model fits to regular pairing data (green) and the prediction for irregular spike-pairs (brown) are taken from Figure 3.

In conclusion, irregular spiking acts in the same direction than increasing firing rate, since irregular spiking patterns combined with a slight increase of frequency rate promotes LTP expression in an extended Δt range, associated with a lack of LTD expression. The model prediction, based on parameters obtained from fitting regular spike-pair data, and the irregular spike-pair experimental data showed remarkable agreement (Figures 4L and 4M).

### Plasticity is dominantly driven by activity bursts upon increasing firing rate

We then asked whether specific spike patterns in the irregular stimulation protocol dominantly shape the plasticity outcome and the predominance of LTP. We hypothesized that bursts of spike pairs might play such a role, i.e., that temporally clustered spike-pairs have a disproportionate impact on synaptic plasticity as previously shown in rat somatosensory and visual cortex, and mice hippocampus.^33^^,^^35^^,^^39^^,^^60^^,^^61^^,^^62^ An alternative hypothesis is that all spike-pairs contribute equally to the induced plasticity, irrespective of their temporal organization within the spike train. In this case, plasticity would be determined solely by the total number of spike-pairs and not by their grouping into bursts. Thus, these two hypotheses make distinct predictions: either bursts dominantly drive plasticity, or plasticity scales proportionally with the number of spike-pairs independent of their temporal structure. To evaluate and compare these hypotheses, we divided the stimulation protocol into bursts and individually occurring spike-pairs (Figures 4A–4C). We defined as bursts all spike-pairs occurring within 150 ms of each other, a timescale relevant for synaptic changes at low rates (Figure 2). All spike-pairs not occurring in bursts, together with the first spike-pair of each burst, formed the alternative “individual pair” stimulation (Figure 4A). The number of bursts and of spikes per burst increase with the stimulation rate as illustrated in the Figures 4B and 4C for irregular stimulation rate from 1 to 4 spikes/second. For irregular stimulation following Poisson statistics, ∼25% of all spikes occur in ∼12 bursts at 1 spike/second and ∼60% of the spikes in ∼28 bursts at 3 spikes/second (Figure 4B). Equivalently, the number of bursts with more than 2 spikes increases with the stimulation rate, but remain below six spikes even at 4 spikes/second (Figure 4C).

Using *burst* and *individual* pair stimulation protocols separately, we first predicted synaptic changes from our model. We then applied the burst protocol experimentally at the cortico-striatal synapse, and compared it to plasticity occurring with the complete irregular stimulation protocols.

**Figure 5:**
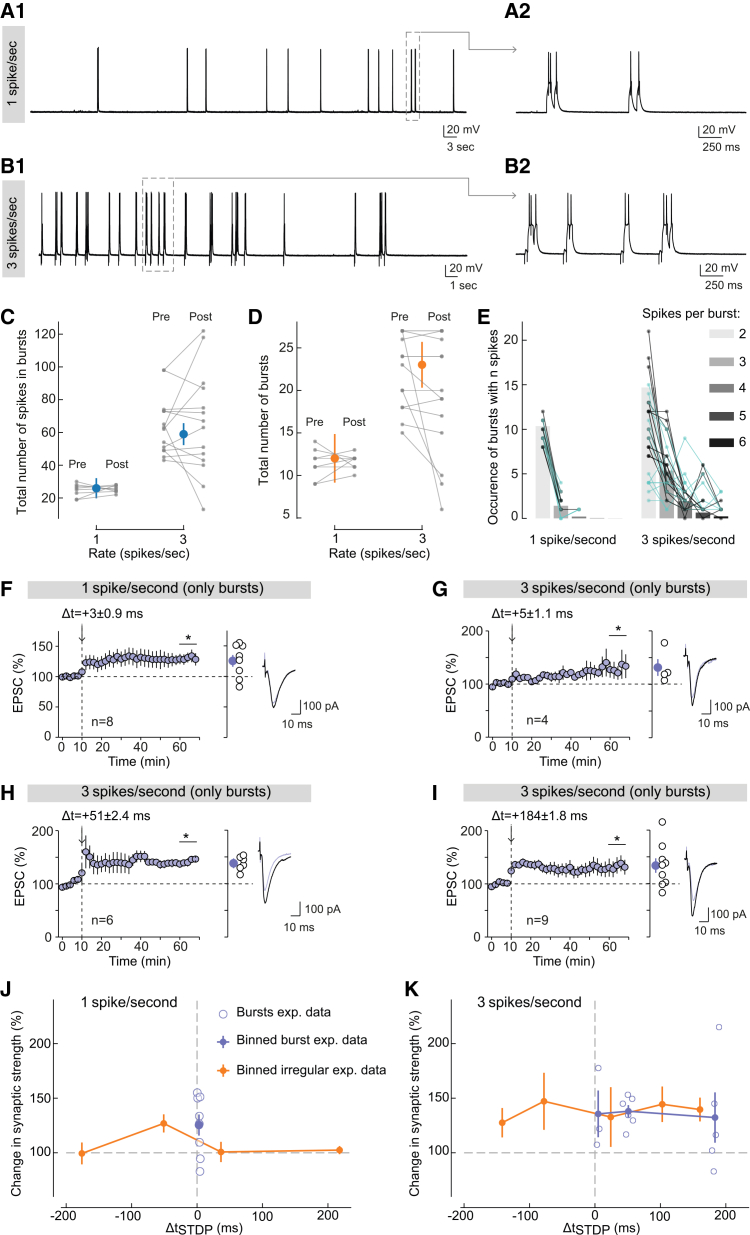
Plasticity is dominantly driven by activity bursts with increasing firing rate (A) Postsynaptic membrane potential recording during an STDP protocol with burst stimulation, containing only spike-pairs occurring within 150 ms of each other, derived from irregular 1 spike/second stimulation. Zoom-in on 2.5 s of the recording (A2). (B) Illustration of burst stimulation generated from 3 spike/second irregular spike-pair stimulation. Zoom-in (B2). Note the higher number of bursts in the same interval in (B) compared to (A). (C and D) Total number of spikes in burst stimulation protocol (C), and total number of bursts (D) for the two stimulation rates used in the experiments, 1 and 3 spikes/second. Each connected pair shows the presynaptic (left) and postsynaptic (right) activity quantification of individual experiments. The theoretical value for Poisson spike trains is shown in blue and orange (mean ± SD). (E) The total number of spikes occurring in bursts for 1 and 3 spikes/second stimulation. Connected points depict the statistics of an individual experiment for pre- (black) and post-synaptic spikes (cyan). (F) Averaged time-courses of synaptic weights after *f* = 1 Hz post-pre irregular burst pairings (Δt = −+3 ± 0.9 ms: 127.6 ± 9.1%, *p* = 0.0297, *n* = 8). (G–I) Averaged time-courses of LTP after *f* = 3 Hz pre-post irregular burst pairings (G, Δt = +5 ± 1.1 ms: 133.9 ± 10.6%, *p* = 0.0237, *n* = 4; H, Δt = +51 ± 2.4 ms: 138.0 ± 5.6%, *p* = 0.0011, *n* = 6; I, Δt = −176 ± 0.9 ms: 134.6 ± 15.5%, *p* = 0.0430, *n* = 9); ∗: *p* < 0.05 by one sample *t* test. (J) Comparison of the experimentally measured synaptic change for pre-post bursts with irregular spike-pairs stimulation experiments as a function of Δt for 1 spike/second stimulation. Individual experimental data points (open purple points) and the average data (full purple point, mean ± SD) summarize the experiments in (F). The binned experimental data or irregular spike-pair stimulation is shown in orange (same as in Figure 4L). (K) Same depiction as in (J) but for 3 spikes/second pre-post burst-paired stimulation (light purple). Experimental data from irregular spike-pair stimulation shown in orange (same as in Figure 4M).

We first examined *in silico* synaptic weight changes for burst versus individual pair stimulation generated from irregular spike-pair stimulation (Figures 4D and 4E). The model predicts that synaptic plasticity induced by individual spike-pair stimulation at 1 spike/second approaches the fit of the model for regular spike-pair plasticity; this is illustrated by the superimposed light purple (individual spike-pairs) and green (fitting of the experimental data using regular spike-pair stimulation) lines in the Figure 4D. Both cases correspond to stimulations with little interactions between consecutive spike-pairs, i.e., >150 ms. At 3 spikes/second stimulation, LTP induced by the irregular spike-pair stimulation protocol can be recovered by using only bursts stimulation, both in terms of potentiation magnitude and induction domain across different time lags Δt; this is illustrated by the dark purple (bursts) and brown (predicted irregular spike-pair stimulation) lines in the Figure 4E. This model behavior is remarkable as only about 60% (Figure 4B) of the spikes are contained in the bursts, compared to the irregular spike-pair protocol, but the same amount of potentiation is induced. Importantly, this reduction in spike rate was not compensated for in either the simulations or the experiments (Figure 5). As a consequence, both the effective stimulation rate (e.g., 3 spikes/second burst stimulation corresponds to ∼1.8 spikes/second) and the total number of spike pairings are reduced proportionally, indicating that bursts induce the same level of LTP at a substantially lower overall rate and spike number. Interestingly, contributions from burst and individual spike-pairs add in a highly non-linear fashion to yield plasticity for the combined irregular pair stimulation protocol, which is the case for both 1 and 3 spikes/second stimulation.

Next, we experimentally tested the model predictions (Figure 5). For this purpose, we applied irregular pairings, conserving only the bursts at 1 or 3 spikes/second (at least two spikes within a 150 ms interval), and thus excluding isolated spikes (*Methods*) (Figures 5A and 5B). We first verified whether the imposed activity pattern of pre- and post-synaptic spikes are in accordance with the theoretical expected values (i.e., the theoretical value for Poisson spike trains) in terms of number of spikes and bursts (Figures 5C and 5D). Presynaptic stimulation was evoked by extracellular synaptic stimulation leading to a reliable induction of postsynaptic EPSPs following irregular pattern (Figures 5C–5E). Postsynaptic spikes were induced through current injections of fixed amplitude and duration in SPNs during the protocol. In turn, refractory period, finite membrane potential rise time and natural fluctuations led altogether to a larger variability in number of generated postsynaptic spikes as expected (Figures 5C–5E). Note that the reduction in spike rate resulting from the removal of isolated spikes for burst-only stimulation was not compensated for, resulting in a reduced effective stimulation rate and a proportional decrease in the total number of spike pairings (see previous paragraph; Figure 4B).

At 1 spike/second, burst stimulation induced LTP for Δt ∼0 ms (Δt = +3 ± 0.9 ms: *p* = 0.0297, *n* = (8) (Figures 5F and 5J). At 3 spikes/second, LTP was expressed after irregular paired bursts at all tested Δt (Δt = +5 ± 1.1 ms: *p* = 0.0237, *n* = 4; Δt = +51 ± 2.4 ms: *p* = 0.0011, *n* = 6; Δt = +184 ± 1.8 ms: *p* = 0.0430, *n* = (9) (Figures 5G–5I and 5K). We observed no difference in the LTP amplitude between the three Δt values tested. Importantly, the LTP amplitude matches with the plasticity obtained for irregular spike-pair stimulation, i.e., a stimulation protocol, which contains 40 times more spike-pairs occurring in isolation.

Overall, the experimental results for plasticity expression with irregular paired-bursts are in full agreement with the model predictions, and underlined the key role for bursts for plasticity induction with increasing firing rate, when compared to single paired-spikes.

## Discussion

Hebbian plasticity and in particular STDP depends on pre- and post-synaptic paired activity (neuronal excitability, Δt, number and frequency of spike-pairs),^3^^,^^4^^,^^6^^,^^15^ but also on third factors, namely neuromodulators, hormones, neurotrophins, peptides and glial cells,^61^^,^^63^^,^^64^^,^^65^^,^^66^ metabolic internal states^67^ and body-brain feedback.^23^ Here, we aimed to identify how parameters of *in vivo*-like irregular firing shape the domain of Hebbian plasticity expression. To investigate synaptic changes elicited by irregular firing at different rates, we used model-driven plasticity experiments at the cortico-striatal synapse. The parameters of model were fitted to synaptic plasticity results obtained with regular spike-pairs at different frequency (Figure 1). We then explored two firing patterns: (i) irregular spike-pairs at different rates; and (ii) irregular spike-pair stripped of individually occurring pairs, leaving bursts of pre-post pairs. For each case, we predicted synaptic modifications induced by changes in firing rate for a stimulation with a fixed number of spike-pairs. We then applied such irregular firing patterns in acute brain slice experiments and determined plastic changes for representative rates and time-delays between pre- and postsynaptic spikes. We observed that irregular spike-pairs at different rates exert similar effects on plasticity expression than the overall frequency increase, i.e., from a progressive Δt broadening of STDP expression domain to a loss of temporal contingency expected for Hebbian synaptic plasticity. Shifting from regular to irregular spike-pairs boosted the effect of the increasing frequency in STDP, since similar plasticity was obtained with irregular spike air at 3 Hz or regular spiking at 10 Hz.

Irregular spiking is composed of bursty and isolated spikes. We found that bursts convey most of the effect of the irregular spike-pair effects in STDP expression. Experimentally, it has been shown that STDP depends mainly on three parameters defining the structure of activity patterns on either synaptic side,^4^^,^^6^ which are the relative timing between pre- and postsynaptic spikes,^10^^,^^11^^,^^12^^,^^13^^,^^14^ the number of spike-pairs^32^^,^^33^^,^^34^^,^^38^^,^^39^ and their frequency.^6^^,^^9^^,^^32^^,^^33^^,^^45^^,^^54^ We observed that the STDP sensitivity to spike-timing could be erased by increasing spike frequency (5–10 Hz in brain slices) and irregular spike-pairs, thus defining the domain of expression of STDP versus a rate-based plasticity. Note that the alteration of a regular activity pattern, such as a low-frequency stimulation (900 stimulations at 1 Hz), with Poisson-distributed interstimulus intervals (to approximate *in vivo*-like patterns) prevents cortical LTD in adult guinea pig brain slices.^68^ Interestingly, acting on firing regularity/irregularity and rate have a similar effect than acting on the glutamate re-uptake by astrocytes, i.e., shifting from Hebbian bidirectional STDP to non-Hebbian LTP, in rat brain slices.^44^ Therefore, this study should help to delineate the condition of emergence of STDP *in vivo*.

In rodent brain slices, various attempts were made to assess the robustness of STDP in naturalistic conditions by complexifying the activity patterns from spike pairs to triplets or quadruplets^24^^,^^27^^,^^33^^,^^69^^,^^70^ or even by replaying *in vivo* spiking patterns as cell conditioning paradigm *ex vivo*.^24^^,^^25^^,^^27^^,^^71^^,^^72^ The classical spike-timing based models capture pair-based STDP,^73^^,^^74^^,^^75^ but do not account for the firing rate-dependence of plasticity (but see Izhikevich and Desai^22^). To address this limitation, more recent models have introduced triplet STDP rules, which show a correspondence with the Bienenstock-Cooper-Munro (BCM) learning rule^76^ and better describe natural stimuli than pair-based STDP.^77^^,^^78^^,^^79^ Interestingly, a triplet-based STDP rule improves the learning capabilities compared to pair-based STDP, when tested at memristive devices and synapses (memristors).^80^^,^^81^ STDP-like protocols were tested *in vivo* in adult cats and rats, and gave rise to bidirectional plasticity as observed *ex vivo*, by synchronizing natural spiking with time-locked sensory stimulation.^19^^,^^20^^,^^82^^,^^83^^,^^84^ Recently, during learning of lever press task in mice, without artificial triggering of spiking activity, STDP mechanisms have been observed *in vivo* at proximal dendrites of layer 2/3 pyramidal cells of the motor cortex.^26^

Classically, STDP-LTP is induced with 100–150 paired stimulations at low frequency (1–2 Hz). Nevertheless, various forms of STDP aiming at mimicking naturalistic activity^15^ were induced with smaller number of pairings (5–30) *ex vivo* in rodents,^33^^,^^34^^,^^36^^,^^38^^,^^39^ as expected in single-trial or one-shot learning.^85^ In particular, at Schaffer collateral-CA1 synapses, an N-methyl-D-aspartate receptor (NMDAR)-mediated LTP is induced with six pairings, while one presynaptic spike paired with a postsynaptic burst of four spikes induced also an LTP but involving α-amino-3-hydroxy-5-methyl-4-isoxazolepropionic acid (AMPA) and ryanodine receptor signaling.^39^ In the same vein, the cortico-striatal NMDAR-mediated LTP required at least 50 pairings to be expressed, but when decreasing the number of pairings down to 5–15 another form of LTP could be induced but relying on the endocannabinoid system.^34^^,^^36^^,^^38^ Overall, the number of pairings as well as the activity pattern on either side of the synapse shape the domain and nature of plasticity expression.

Classically, STDP pairings are performed at 1 or 2 Hz and reported generally bidirectional plasticity which could be asymmetric (t-LTP and t-LTD) or symmetric (only t-LTP or t-LTD).^4^^,^^6^ We chose to perform our experiments in this frequency range starting with pairings at 1 Hz which in the low range of the cortico-striatal activity in awake rats (see paragraph below regarding *in vivo* activity rate in awake rodents). Nevertheless, there are also studies using 0.1, 0.2, or 0.5 Hz pairing protocol reporting bidirectional STDP generally in young mice (<P20)^54^^,^^86^^,^^87^ and some reporting a lack of plasticity (t-LTD disappearing for *p* > 23 in hippocampus)^88^ or unidirectional plasticity (t-LTD at 0.1 Hz).^9^ Increasing pairing frequency has a strong impact on STDP expression.^9^^,^^11^^,^^32^^,^^45^^,^^54^^,^^55^ Namely, increasing the pairing frequency favors LTP whereas low frequency rate favors LTD, in full accordance with the standard BCM plasticity function.^76^ Since, this frequency effect could depend on the developmental stage, it remains to explore early developmental stages to investigate whether irregular spike pairs and burst would have similarly promote LTP.

In the present study, we have explored pairings rate in line with those reported *in vivo* in awake rodents which is in the 1–5 Hz range in the sensory-motor cortex^17^ and 1–6 Hz range in the dorsal striatal^56^^,^^57^^,^^58^^,^^59^ depending on the rat activity (3.3 ± 3.5 Hz in Mahon et al.^57^; 1.4 ± 1.3 Hz in Hawking et al.^58^). Interestingly, SPNs belonging to the direct or indirect *trans*-striatal pathways both show similar average firing rate.^59^ However, if in average SPNs fire at relatively low frequency, the low limit of the range sits at 0.1 Hz (full range for active SPNs, [0.1–10.8] Hz in Mahon et al.^57^). It thus remains to explore such very low regimes of activity to evaluate whether the impact of irregular spike pairs and burst would have similar effects than at higher frequency rates (1–5 Hz).

It should be emphasized that the Δt broadening (upon increasing frequencies and/or irregular activity) does not imply that STDP would be replaced by another form of plasticity, e.g., a rate-based plasticity, but indicates an accrued probability of occurrence of STDP induction. An essential characteristic of STDP is its expression for matched activities occurring within a restricted time window. As a result, decorrelated events (>30–50 ms in most cases in rodents) fail to trigger plasticity. This view is only valid when considering single spike activities, since we show here that bursty activities would allow decorrelated information to be bound and give rise to plasticity. Therefore, bursty activity (at a low and medium frequency) would considerably extend the domain of STDP expression to behavioral timescales,^89^ and possibly fill the gap between STDP *stricto sensu* and behavioral timescale synaptic plasticity (BTSP).^37^^,^^41^^,^^90^ BTSP relies on millisecond-second scale calcium dendritic plateau paired with presynaptic activity occurring within a 5–10 s range, which remains shorter than STDP involving multiple pairings (at 1 or even 3 Hz). Nevertheless, STDP with bursty activity patterns should allow reducing the total duration of the conditioning activity needed for plasticity induction, as well as a Δt broadening. In this line, STDP involving regular spike-pairs at low frequency should therefore be viewed as a safety measure to ensure that activities involving few spikes do not systematically produce synaptic plasticity, and that synaptic plasticity can only occur during regular activity and within a restricted time window. The restricted timescale of STDP (typically <50–60 ms) is already sufficient to account for *in vivo* learning, as exemplified for replay episodes in hippocampus during sleep (e.g., the modeling study by George et al.^91^). In addition, variations of neuromodulator concentrations (e.g., dopamine, acetylcholine, serotonin) can considerably enlarge the Δt STDP and this without change of the spike-pair pattern activity.^63^^,^^65^^,^^66^ Therefore, irregular firing and bursty activity in combination with the third factors allow STDP to fulfill behavioral temporal requirement for plasticity induction and learning. In addition, the plasticity expression reported here in juvenile (P25-35) mice, although this remains to be confirmed in adult mice, is expected to be robust in older mice. Indeed, at cortico-striatal synapses, the major developmental difference occurs in younger animals (<P10) with unidirectional Hebbian STDP-LTD expression, whereas for older ages (starting from P15, and verified for both pre-adult P30-60, and adult, P60-90) a bidirectional anti-Hebbian STDP could be reliably induced *ex vivo* in rats and mice^44^^,^^45^ and *in vivo* in adult rats.^50^^,^^92^ STDP is a multifaceted phenomenon involving various signaling pathways and rules depending on the neuronal subpopulations, brain structures and/or developmental stages.^6^ To generalize (or not) our experimental data and model predictions it remains to explore whether such observations apply at various stages of development and other structures, in which it has been shown that when probability of glutamate release is LTD is favored whereas upon maturation the probability of release decreases favoring LTP.^93^^,^^94^^,^^95^ If most of STDP are calcium-based, it remains to explore whether calcium-independent STDP^96^ would follow predictions drawn in the present study.

It remains to determine whether this cortico-striatal STDP sensitivity to frequency and irregular spike-pairs is similar *in vivo*, in other brain areas, along development or even in other species, for which STDP features strongly differ.^6^^,^^7^^,^^66^ Indeed, at the same cortico-striatal synapses, we have reported that an *in vivo* STDP protocol (regular spike-pairs at 1 Hz repeated 100 times) in anesthetized adult rats induced bidirectional plasticity as reported here, but in a much larger temporal window of ∼200 ms,^50^ in the same range that in the visual cortex of juvenile rats.^20^ Similarly, STDP temporal window was wider, i.e., ∼150–200 ms, in hippocampal pyramidal cells in human biopsies slices, than in rodents.^28^ Broadening of the STDP temporal window can arise from an increased jittering between pre- and postsynaptic element as observed in the rat visual cortex.^20^ Irregular spiking and frequency can also have distinct effects depending on the underlying signaling pathway. Indeed, with increasing spike timing jitter (mimicking noisy inputs) the NMDA-mediated STDP appears more fragile than the endocannabinoid-mediated plasticity.^97^ It should also be interesting to explore whether irregular spike-pairs, rate and bursty activity rules as characterized here also apply to the input-timing dependent plasticity^98^ viewed as a more physiological form of STDP and dependent upon the temporal correlation between two distinct presynaptic afferences, i.e., without artificial postsynaptic current manipulation.^99^ Naturalistic STDP and associated expressed plasticity could also be of importance in field of memristive synapses and artificial neural networks implemented with brain-inspired STDP^15^^,^^80^^,^^100^ to extend the performance of neuromorphic computing.

Overall, our results identify the dominance of some key components of the activity pattern (irregular over regular spike-pairs, bursts over isolated spikes) governing the temporal window extent and STDP polarity, and delineate the domain of expression of Hebbian plasticity. The strong impact of naturalistic patterns, such as irregular and/or bursty firing, considerably extend the domain of expression of STDP and bridges the gap with behavioral timescales.

### Limitations of the study

For a generalization of our observations, it remains to determine whether similar rules would apply to other forms of STDP such as asymmetric Hebbian STDP and symmetric anti-Hebbian or Hebbian STDP, or calcium-independent forms of STDP, to various neuronal subpopulations (GABAergic interneurons), other cerebral structures (than dorsolateral striatum) or early developmental stages (not explored in this study).

## Resource availability

### Lead contact

Request for further information should be directed to and will be fulfilled by the lead contact, Laurent Venance (laurent.venance@college-de-france.fr).

### Materials availability

All data (electrophysiology and modeling) are available in the main text or the supplementary materials.

This study did not generate new unique reagents.

### Data and code availability

• All data reported in this study will be shared by the lead contact upon request
• All original code has been deposited at Github and is publicly available at https://github.com/mgraupe/CalciumPlasticityModelIrregularActivity/tree/venanceData as of the date of publication.
• Any additional information required to reanalyze the data reported in this study is available from the lead contact upon request.

## Acknowledgments

We thank the members of the L.V. laboratory for helpful suggestions and critical comments.

This work was supported by fundings from 10.13039/501100001677Inserm, 10.13039/501100004794CNRS, 10.13039/501100021804Collège de France.

S.V. was a Research Fellow of the MRT and the LabEx MemoLife. S.O. was supported by the program Ecoles Universitaires de Recherche launched by the French Government and implemented by the 0041NR, with the reference ANR-17-EURE-0017. This work has received support under the Major Research Program of PSL Research University PSL-Neuro launched by 10.13039/501100009517PSL Research University and implemented by ANR (ANR-10-IDEX-0001).

## Author contributions

Conceptualization, L.V., M.G., and S.O.; whole-cell patch-clamp recordings and analysis, Y.D., S.V., Y.C., and Z.Z.; conception and the design of the mathematical model, M.G. and S.O.; acquisition and analysis of data from the mathematical model, M.G. and S.O.; writing of the manuscript, M.G. and L.V. wrote the manuscript and all authors have edited and corrected the manuscript; funding acquisition, L.V. supervision: L.V. and M.G.

## Declaration of interests

The authors declare no competing interests.

## STAR★Methods

### Key resources table

**Table:** 

REAGENT or RESOURCE	SOURCE	IDENTIFIER
**Experimental models: Organisms/strains**		

Rat Sprague Dawley	Charles River	400

**Software and algorithms**		

Patchmaster v2x32	Heka Elektronik	https://www.heka.com/products/products_main.html#soft_pm
Fitmaster	Heka Elektronik	https://www.heka.com/products/products_main.html#soft_fit
Igor-Pro 6.0.3	Wavemetrics	https://www.wavemetrics.com/products/igorpro
MATLAB R2012b	Mathworks	https://fr.mathworks.com/products.html?s_tid=gn_ps
Prism 5.02	GraphPad	https://www.graphpad.com/scientific-software/prism/
Python 3.9.18.	Anaconda Software Distribution	https://www.anaconda.com/products/individual
Original code for the mathematical model	This paper	https://github.com/mgraupe/CalciumPlasticityModelIrregularActivity/tree/venanceData
Illustrator 2026 30.1	Adobe	https://www.adobe.com/fr/

**Other**		

Vibrating blade microtome	Leica	VT1200S
Patch-clamp amplifiers	Heka Elektronik	EPC10-2, EPC10-4
Acquisition interface	Heka Elektronik	LIH8+8
Temperature Controller	Luigs&Neumann	Bath-controller model V
Stimulus Isolator	AMPI	Iso-Flex
Stimulus generator	AMPI	Master 9
Concentric bipolar electrode (for stimulation)	Phymep	CBDSF75
Patch-clamp glass-thin walls (CEI)	Phymep	PG150T-75
Motorized manipulators	Luigs&Neumann	LN-junior

### Experimental model and study participant details

#### Animals

All experiments were performed in accordance with the guidelines of the local animal welfare committee (Center for Interdisciplinary Research in Biology Ethics Committee) and the EU (directive 2010/63/EU). Center for Interdisciplinary Research in Biology agreement number: D750512 for the animal experimentation. Every precaution was taken to minimize stress and the number of animals used in each series of experiments. Pre-adult male Sprague-Dawley rats P25-35 (Charles River, L’Arbresle, France) were used for acute brain slice electrophysiology. Animals were group housed in standard 12-h light/dark cycles and food and water were available *ad libitum*.

### Method details

#### Brain slice preparation and patch-clamp recordings

Horizontal brain slices (300 μm-thick) containing the somatosensory cortical area and the corresponding cortico-striatal projection field in the dorsolateral striatum^43^^,^^45^ were prepared with vibrating blade microtomes (Microtome 7000smz-2, Campden Instruments Ltd., UK; VT1000, Leica Microsystems, Nussloch, Germany). Cortico-striatal connections (between somatosensory cortex layer 5 and the dorsolateral striatum) are preserved in the horizontal plane. Brains were sliced in a 95% CO2/5% O2-bubbled, ice-cold cutting solution containing (in mM) 125 NaCl, 2.5 KCl, 25 glucose, 25 NaHCO3, 1.25 NaH2PO4, 2 CaCl2, 1 MgCl2, 1 pyruvic acid, and transferred into the same solution at 34°C for 1 h and next moved to room temperature. whole cell recordings were performed as previously described.^45^^,^^46^ For whole cell recordings, borosilicate glass pipettes of 5–7 MΩ resistance were filled with (in mM): 105 K-gluconate, 30 KCl, 10 HEPES, 10 phosphocreatine, 4 Mg-ATP, 0.3 Na-GTP, 0.3 EGTA (adjusted to pH 7.35 with KOH). The composition of the extracellular solution was (mM): 125 NaCl, 2.5 KCl, 25 glucose, 25 NaHCO3, 1.25 NaH2PO4, 2 CaCl2, 1 MgCl2, 10 μM pyruvic acid bubbled with 95% O2 and 5% CO2. All recordings were performed at 34°C, using a temperature control system (Bath-controller V, Luigs & Neumann, Ratingen, Germany) and slices were continuously superfused with extracellular solution, at a rate of 2 mL/min. Slices were visualized under an Olympus BX51WI microscope (Olympus, Rungis, France), with a 4×/0.13 objective for the placement of the stimulating electrode and a 60×/1.00 water-immersion objective for the localization of cells for whole cell recordings. Signals were amplified using EPC10-2 amplifiers (HEKA Elektronik, Lambrecht, Germany). Current- and voltage-clamp recordings were sampled at 10 kHz with the Patchmaster v2x32 program (HEKA Elektronik).

#### Spike-timing-dependent plasticity induction protocols

Electrical stimulations were performed with a concentric bipolar electrode (Phymep, Paris, France) placed in layer 5 of the somatosensory cortex. Electrical stimulations were monophasic, at constant current (ISO-Flex stimulator, AMPI, Jerusalem, Israel). Currents were adjusted to evoke 100–300 pA EPSCs. Repetitive control stimuli were applied at 0.1 Hz.

##### Spike-timing-dependent plasticity with regular stimulation patterns

STDP protocols consisted of pairings of pre- and postsynaptic stimulations separated by a specific time interval (Δt). Presynaptic stimulations corresponded to cortical stimulations and the postsynaptic stimulation of an action potential evoked by a depolarizing current step (30 ms duration) in SPNs. The STDP protocol involved pairing pre- and postsynaptic stimulation with a certain fixed timing interval, Δt (Δt<0 indicating that postsynaptic stimulation preceded presynaptic stimulation, i.e., post-pre pairings, and Δt>0 indicating that presynaptic stimulation preceded postsynaptic stimulation, i.e., pre-post pairings), repeated *n* times at 1, 2.5–3, 5 or 10 Hz. For regular paired stimulation patterns, we investigated the outcome of STDP at various fixed Δt with regular inter-pair intervals. Voltage-clamp recordings were made over a period of 10 min at baseline, and for at least 40 min after the STDP induction protocols; long-term changes in synaptic weight were measured for the last 10 min. We individually measured and averaged 60 successive EPSCs, comparing the last 10 min of the recording with the 10-min baseline recording. Access and input resistance were measured every 10 s during the experiment and recordings were discarded if access resistance changed by more than 20%.

##### Spike-timing-dependent plasticity with irregular stimulation patterns

For irregular stimulation patterns we investigated the outcome of STDP at various fixed Δt with irregular inter-pair intervals distributed pseudo-randomly accordingly to a Poisson distribution.

##### Spike-timing-dependent plasticity with burst stimulation patterns

For burst stimulation patterns we extracted stimulating patterns from irregular stimulation where all spike-pairs occurring more than 150 ms apart from each other were removed and effectively kept only bursts occurring within 150 ms.

The *irregular*- and the *burst* stimulation protocols for each recorded pair was generated anew using a pseudo-random Poisson process. Thus, each experiment used a distinct realization of the stimulation pattern with identical statistical properties (same mean firing rate and Δt).

#### Electrophysiological data analysis

Offline analysis was performed with Fitmaster (Heka Elektronik), Igor-Pro 6.0.3 (Wavemetrics, Lake Oswego, OR, USA) and custom-made software in Python 3.0. Statistical analysis was performed with GraphPad Prism 5.02 software (San Diego, CA, USA). All results are expressed as mean ± SEM. Statistical significance was assessed by two-tailed student t-tests (unpaired or paired t-tests), using the indicated significance threshold (*p*). In all cases “n” refers to an experiment on a single cell from a single brain slice.

#### Calcium-based model of synaptic plasticity

We theoretically investigated plasticity using the calcium-based model where the postsynaptic calcium concentration determines the temporal evolution of the synaptic weight variable, *w*. The postsynaptic calcium in turn is a function of pre- and postsynaptic activity. The model is studied extensively in Graupner and Brunel^42^ and Graupner et al.^31^).

Shortly, the postsynaptic calcium concentration drives changes in ρ according

(Equation 1) $$\tau \dot{\rho }=-\rho \left(1-\rho \right)\left(1/2-\rho \right)+\gamma_{\rho }\left(1-\rho \right)\Theta \left[c\left(t\right)-\theta_{p}\right]-\gamma_{d}\rho \Theta \left[c\left(t\right)-\theta_{d}\right]+Noise\left(t\right)\text{,}$$

is the time constant of the synaptic efficacy changes happening on the order of seconds to minutes. The first term on the right-hand side describes the dynamics of *w* in the absence of pre- and postsynaptic activity. The cubic function endows the synapse with bistability, with stable states at ρ = *0* and at ρ = *1*. The second and third terms on the right-hand-side of Equation 1 describe in a simplified fashion calcium-dependent signaling cascades leading to synaptic potentiation and depression, respectively. The synaptic efficacy variable tends to increase, or decrease, when the instantaneous calcium concentration, c(t), is above the potentiation $\theta_{p}$ or the depression threshold $\theta_{d}$, respectively (Θ denotes the Heaviside function, Θ[c−θ]=0 for c<θ and Θ[c−θ]=1 for c≥θ). The parameter $\gamma_{p}$ (resp. $\gamma_{d}$) measures the rate of synaptic increase (resp. decrease) when the potentiation (resp. depression) threshold is exceeded. The last term in Equation 1 is an activity dependent noise term $\left(t\right)=\sigma \sqrt{T\Theta }\left[c\left(t\right)-\min\left(\theta_{d},\theta_{p}\right)\right]\eta \left(t\right)$ , where σ is the amplitude of the noise, η(t) is a Gaussian white noise process with unit variance density, and the Heaviside function Θ gives an activity dependence to noise. See Graupner and Brunel^42^ for more details, and Table 1 for parameters.

The postsynaptic calcium dynamics are described by:

(Equation 2) $$c\left(t\right)=\sum_{i}C_{pre}\;\exp\left(-\frac{t-t_{i}-D}{T_{Ca}}\right)+\sum_{j}C_{post}\;\exp\left(-\frac{t-t_{j}}{T_{ca}}\right)$$

where c is the total calcium concentration, $T_{ca}$ the calcium decay time constant, $C_{pre}$ and $C_{post}$ the pre- and postsynaptically evoked calcium amplitudes. The sums run over all pre- and postsynaptic spikes occurring at times $t_{i}$ and $t_{j}$ respectively. The time delay, *D*, between the presynaptic spike and the occurrence of the corresponding postsynaptic calcium transient accounts for the slow rise time of the NMDAR-mediated calcium influx. Without loss of generality, we set the resting calcium concentration to zero, and use dimensionless calcium concentrations. The parameters of the model were fitted to the experimental data (see below “Fitting the calcium-based plasticity model” and Table 1 for parameter values).

#### STDP stimulation protocols

To study changes in synaptic efficacy induced by spike-timing relationships and firing rate under different conditions, we generated stimulation protocols, which consisted of spike-pairs with fixed delay, Δt, between pre- and postsynaptic stimulation but varied occurrences:

1 Regular presentation for which intervals between spike-pairs was fixed and determined by the stimulation frequency, f.
2 Irregular presentation for which the occurrence of spike-pairs followed Poisson statistics with rate v.
3 Burst presentation was derived from the irregular presentation by removing all spike-pairs that occurred more than 150 m s apart from each other, and keeping bursts occurring within 150 m s.
4 Individual pair presentation was again derived from the irregular presentation but only spike-pairs occurring more than 150 m s apart from each other were kept. Additionally, all first spike-pairs occurring in a burst were included in that protocol (see Figure 4A for an illustration).

All stimulation protocols consisted of 100 spike-pairs. Throughout this study, we systematically varied the delay between pre- and postsynaptic spike, Δt, and the frequency (f) or rate for irregular occurrence (*v*) at which spike-pairs were presented.

#### Fitting the calcium-based plasticity model

For our combined modeling and experimental approach, we fitted the calcium-based model to regular spike-pair data. Our fits included experimental plasticity datasets for 1, 3, 5, and 10 Hz stimulation. We required that the same parameter set reproduces all conditions, modifying only the spike-pair presentation frequency, *f* (Figures 1H–1K). 9 parameters of the model were optimized during the fitting routine (Table 1). Fixed parameters were the calcium thresholds $\theta_{d}$ and $\theta_{p}$ as well as the fraction of synapses in the DOWN state, β. Calcium amplitudes and thresholds are scaled by $\theta_{d}$ (i.e., $\theta_{d}=1$) without loss of generality. The value of $\theta_{p}$ was determined based on initial fit results at 1.5. It was fixed to stabilize the highly non-linear fitting routine. We assume that, before a stimulation protocol, a fraction β = 0.5 of the synapses are in the DOWN state.

We defined the goodness of fit to the experimental data by a cost function which is the sum of all squared distances between binned data points (full black circles in Figures 1H–1K) and the values obtained from the calcium-based model. We drew initial parameter values from a uniform distribution and use the downhill simplex algorithm to search for the minimum of the cost function. The fit is repeated >10^9^ times and the parameter set with the lowest cost function is used (Table 1).

We take the synaptic strength linearly related to was w = w0 + ρ(w1 − w0), where w0 and w1 are the synaptic strengths of the DOWN/UP state. b = w1/w0 is the ratio between the synaptic strengths of the UP vs. DOWN state. We consider the change in synaptic strength as the ratio between the average synaptic strengths after and before the stimulation w(after stim)/w(before stim), *i*.*e*.,:

([(1−U)β+D(1−β)]+b[Uβ+(1−D)(1−β)])/(β+[1−β]b)

with U and D denote the UP and the DOWN transition probabilities computed from the model.^42^

The model is implemented in Python (3.9.18 version), and the scripts for parameter fitting, simulations, and figure generation are publicly available at:https://github.com/mgraupe/CalciumPlasticityModelIrregularActivity/tree/venanceData

### Quantification and statistical analysis

Quantification and statistical analysis were performed with GraphPad Prism 5.02 version. Results are expressed as mean ± SEM. Statistical significance was assessed by two-tailed student t-tests (unpaired or paired t-tests). The level of significance in all figures is illustrated with ∗: *p* < 0.05, with the corresponding exact *p* values in the Figure Legends. In all cases, “n” refers to the number of whole cell recordings on a single cell (SPN) from a single brain slice.

No statistical method was used to predetermine sample size. No data were excluded from the analyses, except SPNs displaying input and series resistances varying by >20%. Specific tests are stated in the figure legend for each of the experiments.
