# Supplementary material for: Irregular and bursty neuronal firing extend spike-timing dependent plasticity window to behavioral timescales
Source: iScience. 2026 Aug 4;29(8):117052. doi: 10.1016/j.isci.2026.117052 (PMC13463992; doi:10.1016/j.isci.2026.117052)
Supplement: Supplementary file 1 [file mmc1.pdf]

iScience, Volume *29*

Supplemental information

Irregular and bursty neuronal firing extend spike-timing dependent plasticity window to behavioral timescales

Yulia Dembitskaya, Silvana Valtcheva, Yihui Cui, Zhiwei Zheng, Srdjan Ostojic, Michael Graupner, and Laurent Venance

Document S1

SUPPLEMENTARY FIGURE 1 and LEGEND

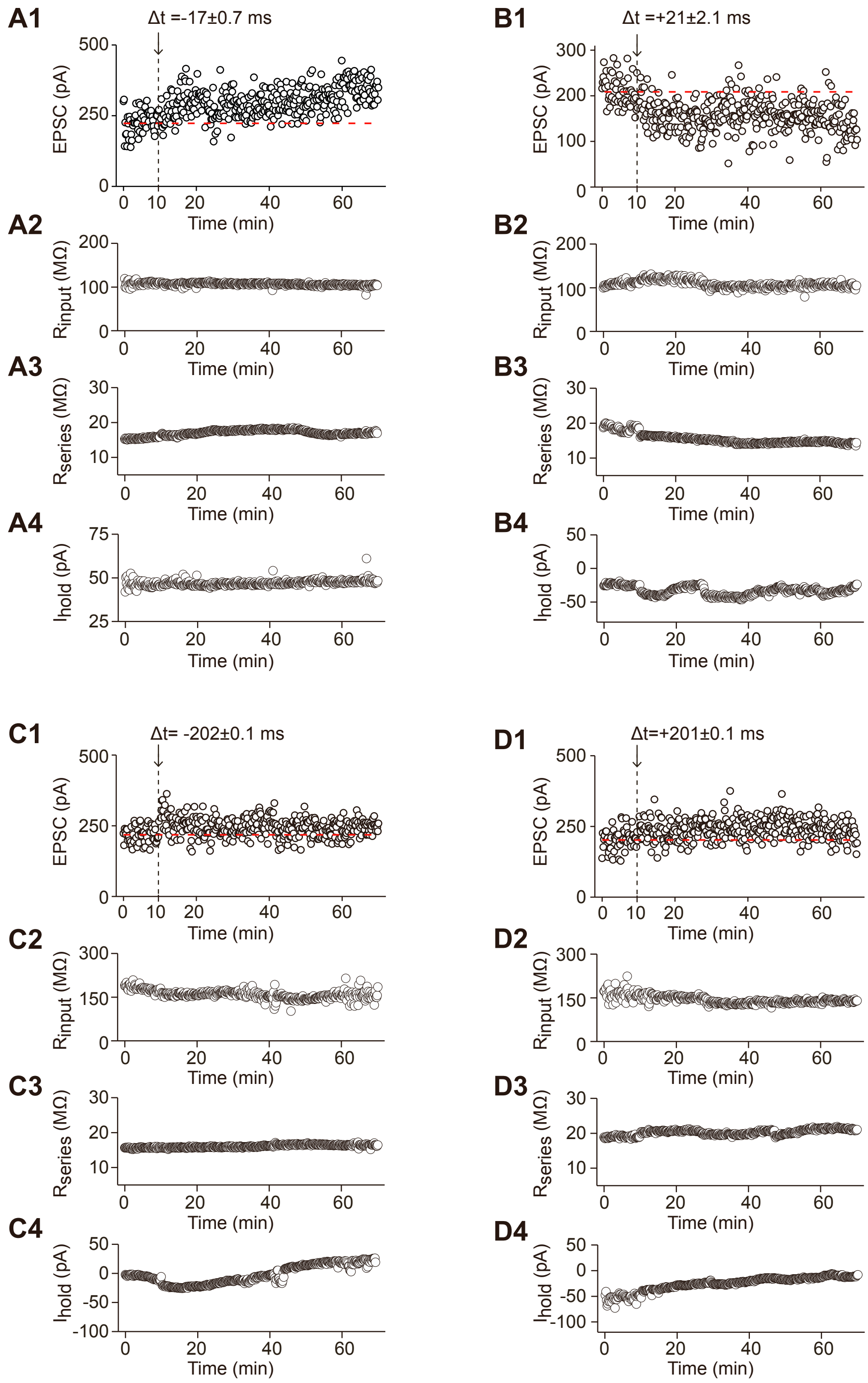

Supplementary Figure 1: Representative STDP for regular spike-pair stimulation (1 spike/second) (related to Figure 1)**.**

(**A** and **B**) Representative cortico-striatal STDP at 1 Hz with Δt_{STDP}~+/-20 ms. (**A**) Example of plasticity induced by 100 post-pre pairings (Δt_{STDP}=-18 ms) (baseline: 235±6 pA, and 50-60 min after pairings: -349±5 pA). Bottom, time course of Ri (baseline: 108.5±0.6 MΩ and 50-60 min after pairings: 103.9±0.4 MΩ), Rs (baseline: 15.5±0.03 MΩ and 50-60 min after pairings: 16.9±0.03 MΩ) and injected current (baseline: +10.5±2.1 pA and 50-60 min after pairings: -22.4±0.2 pA) (**B**) Example of plasticity induced by 100 pre-post pairings (Δt_{STDP}=+21 ms) (the mean baseline EPSC amplitude was 208.7±4.6 pA before pairings and was decreased to 134.9±4.1 pA one hour after pairings). Bottom, time course of Ri

(baseline: 109.2±0.5 MΩ and 50-60 min after pairings: 103.5±0.5 MΩ), Rs (baseline: 18.5±0.09 MΩ and 50-60 min after pairings: 14.2±0.03 MΩ) and injected current (baseline: -24.4±0.2 pA and 50-60 min after pairings: -30.4±0.7 pA). (**C** and **D**) Representative cortico-striatal STDP at 1 Hz with Δt_{STDP}~+/-200 ms. (**C**) Example of plasticity induced by 100 post-pre pairings with Δt_{STDP}=-202 ms (baseline: 218±4 pA, and 244±3 pA, one hour after pairings). Bottom, time course of Ri (baseline: 179.9±1.3 MΩ and 50-60 min after pairings: 156.7±2.2 MΩ), Rs (baseline: 15.6±0.02 MΩ and 50-60 min after pairings: 16.41±0.04 MΩ) and injected current (baseline: -5.8±0.4 pA and 50-60 min after pairings: 19.1±0.7 pA). (**D**) Example of plasticity after 100 pre-post pairings (Δt_{STDP}=+201 ms) (baseline EPSC amplitude was 202±5 pA before pairings and 50 min after pairings, 225±4 pA). Bottom, time course of Ri (baseline: 163.8±4.1 MΩ and 50-60 min after pairings: 139.4±0.4 MΩ;), Rs (baseline: 18.9±0.03 MΩ and 50-60 min after pairings: 21.2±0.04 MΩ) and injected current (baseline: -50.8±0.8 pA and 50-60 min after pairings: -10.0±0.3 pA).
